# Immunomodulatory Effects Mediated by Nano Amorphous Calcium Phosphate/Chitosan Oligosaccharide Lactate Coatings Decorated with Selenium on Titanium Implants

**DOI:** 10.3390/jfb14040227

**Published:** 2023-04-18

**Authors:** Marijana R. Pantović Pavlović, Nenad L. Ignjatović, Vladimir V. Panić, Ivana I. Mirkov, Jelena B. Kulaš, Anastasija Lj. Malešević, Miroslav M. Pavlović

**Affiliations:** 1 Department of Electrochemistry, Institute of Chemistry, Technology and Metallurgy, National Institute of the Republic of Serbia, University of Belgrade, 11000 Belgrade, Serbia; panic@ihtm.bg.ac.rs; 2 Center of Excellence in Chemistry and Environmental Engineering—ICTM, University of Belgrade, 11000 Belgrade, Serbia; 3 Institute of Technical Science of the Serbian Academy of Sciences and Arts, 11000 Belgrade, Serbia; nenad.ignjatovic@itn.sanu.ac.rs; 4 Department of Chemical-Technological Sciences, State University of Novi Pazar, 36300 Novi Pazar, Serbia; 5 Immunotoxicology Group, Department of Ecology, Institute for Biological Research “Sinisa Stankovic”—National Institute of the Republic of Serbia, University of Belgrade, 11000 Belgrade, Serbia; mirkovi@ibiss.bg.ac.rs (I.I.M.); jkulas@ibiss.bg.ac.rs (J.B.K.); anastasija.malesevic@ibiss.bg.ac.rs (A.L.M.)

**Keywords:** deposition, hybrid coating, immunomodulation, implants, inflammatory mediators, nano calcium phosphate

## Abstract

The aim of this work is in situ anodization/anaphoretic deposition of a nano amorphous calcium phosphate (ACP)/chitosan oligosaccharide lactate (ChOL) multifunctional hybrid coating decorated with selenium (Se) on a titanium substrate and in vivo investigation of its immunomodulatory and anti-inflammatory effect. Investigating phenomena at the implant–tissue interface of interest for controlled inflammation and immunomodulation was also the aim of the research. In our earlier research, we designed coatings based on ACP and ChOL on titanium with anticorrosive, antibacterial and biocompatible properties, while in the presented results we show that selenium addition makes this coating an immunomodulator. The immunomodulatory effect of the novel hybrid coating is characterized by the examination of the functional aspects in the tissue around the implant (in vivo): proinflammatory cytokines’ gene expression, M1 (iNOS) and M2 (Arg1) macrophages, fibrous capsule formation (TGF-β) and vascularization (VEGF). The EDS, FTIR and XRD analyses prove the formation of a ACP/ChOL/Se multifunctional hybrid coating on Ti and the presence of Se. A higher M2/M1 macrophage ratio in the ACP/ChOL/Se-coated implants compared to pure titanium implants (a higher level of Arg1 expression) is noted at all time points examined (after 7, 14 and 28 days). Lower inflammation measured by gene expression of proinflammatory cytokines IL-1β and TNF, lower expression of TGF-β in the surrounding tissue and higher IL-6 expression (solely at day 7 post-implantation) is noted in presence of the ACP/ChOL/Se-coated implants.

## 1. Introduction

Titanium (Ti) and its alloys have been widely and successfully used in various fields of medicine and dentistry so far [1]. Titanium-based materials are the basis of many biomedical devices due to their favorable mechanical, biocompatible and physicochemical properties [2]. There are different strategies implemented towards improvement of the properties and applications of titanium implants in medicine and orthopedics. One of the first successful strategies was the coating of implant surfaces with calcium phosphates (CP) and hydroxyapatite (HAp) [3]. Application of HAp coatings on titanium has achieved numerous benefits, primarily in enabling strong interface formation with the natural bone [4]. In the next generation of CP and HAp coatings on titanium surfaces, researchers have directed their investigations towards designing coatings that, in addition to the already known good biocompatible and osteoconductive properties, also possess a new one—antimicrobial, which is also very important for potential clinical application in medicine and dentistry. A special contribution to the further development of CP coatings on titanium was made possible by the use of nanotechnologies with the aim of designing small CP and HAp particles with significantly larger specific surface area, which potentially enables their antimicrobial properties [5,6]. Amorphous calcium phosphate (ACP) proved to be suitable for the deposition on metal substrates by low-temperature wet route or by high-temperature dry route [7]. Electrophoretic deposition (EPD) is one of the most attractive methods for obtaining uniform coatings with controlled microstructure. With this method, it is possible to obtain a coating with the desired properties on substrates of complex shape, of porous structure and at room temperature without the need for expensive process equipment [8,9,10]. There are two types of electrophoretic deposition processes: cataphoretic and anaphoretic. The former deposition process of HAp coatings is well known and explained in the literature [11,12]. The main problem that occurs is the adhesion of the coatings on the substrate, which is usually overcome with sintering. However, sintering process and high temperature can decrease bone integration efficiency [12]. On the other hand, anaphoretic deposition of CPs is a relatively newly presented technique. After anodization/anaphoretic electrodeposition of nano ACP on a Ti substrate, its transformation into HAp as a final coating was determined [13], which can enable even a better osteoconductive potential of this types of coatings in bone tissue engineering. The process of phase transformation of ACP into HAp, which is also registered in living systems, is complex and basically contains processes of recrystallization of ACP into HAp [14,15].

Chitosan (Ch), a linear polysaccharide obtained from sea shrimps, is widely used in the pharmaceutical and food industry, primarily due to its good antimicrobial properties and harmlessness in human nutrition [16]. Chitosan derivatives have also been the subject of extensive in vitro and in vivo research due to their specific antimicrobial properties [17]. By a simple dip-coating procedure, coatings of micro-nano-structured HAp with chitosan on a Ti substrate were obtained, which achieved significant antibacterial properties [18]. In the authors’ previous research, the possibility of obtaining a coating based on nano ACP and Ch derivatives with high adhesion on a Ti substrate using simultaneous anodization/anaphoretic electrodeposition was indicated [13]. By optimizing the anodization/anaphoretic electrodeposition process, the authors designed coatings that, in addition to bioactive and antimicrobial properties, also achieved advanced corrosion-resistant properties important for their potential use in living systems [9]. A. Pawlik et al. also obtained a Ch-HAp coating on a Ti substrate using the electrophoretic deposition method and found that the deposition time and voltage affect the morphology of the coating [19]. Tubular structures based on Ti and Ch obtained by the electrochemical single-step process indicated their high potential in application as dental implants [20]. HAp/Ch-based coatings on Ti substrates have also been shown to be suitable for the immobilization of antibiotics and their local release into the biological environment [21]. In situ electrochemical deposition may be suitable for the immobilization of not only antibiotics in HAp- and Ch-based coatings, but also antimicrobial elements such as silver (Ag) [22].

Implantation of a foreign object in a mammalian body always causes a foreign body response (FBR), regardless of the biocompatibility of the material from which the object is composed [23]. The body’s immune response after reconstruction can completely cancel a successful surgical intervention and a successful choice of materials. There are numerous strategies in designing materials with anti-inflammatory properties. Basic strategies are based on designing materials with intrinsic anti-inflammatory properties, mainly focused on functionalization of the material surface or on releasing anti-inflammatory molecules [24,25]. So far, in the literature and in the field of bone tissue engineering, there is a very small number of published studies that directly present the results of these strategies. In the area of bone tissue reconstruction, the possibility of functionalizing the Ti implant surface with a magnesium/zinc coating was examined in order to immunomodulate and reduce inflammation after implant application [26]. By electrophoretic deposition, a coating based on Ch and dexamethasone, which has anti-inflammatory properties, was successfully designed on a steel substrate [27]. Recent research has also shown that Ch has significant anti-inflammatory activity in addition to anti-microbial properties [28], which is of interest in designing the properties of coatings based on HAp and Ch.

Selenium (Se) is an essential trace element that participates in the regulation of immune functions through the redox-regulating activity of selenoproteins by the mechanism of protecting immune cells from oxidative stress [29]. In addition to the important role of Se in the regulation of inflammatory processes, it also participates in the regulation of immunity [30]. Recent studies have confirmed that Se in small doses participates in processes that are directly related to antitumor immunity [29]. In contrast to the authors’ earlier research, which was directed towards designing a coating based on HAp and Ch derivatives, chitosan oligo lactate (ChOL) on Ti substrates with biocompatible, antimicrobial and corrosion resistant properties [9,12,13,31], our current research is in the field of optimizing the process of obtaining next-generation coatings based on nano ACP, ChOL and Se, which can enable engineered immunomodulation.

According to the International Organization for Standardization (ISO), medical devices/implants might be considered biocompatible if after implantation in soft tissue are found to have a thin fibrous capsule with little evidence of ongoing inflammatory reaction [32]. Consequently, the determination of biocompatibility of materials for potential application in humans is aimed at the histological examination of fibrous capsule thickness and the presence of inflammatory cells at various time points after subcutaneous implantation [33,34,35,36,37,38]. In 2021, a paper has been published that proposed to redefine the term biocompatibility in order to describe materials that lead to vascularized, reconstructive healing and functional tissue integration with little or no fibrosis [39]. Additionally, the authors suggested to take into consideration the activity of various cells that can lead to an inflammatory reaction or tissue reconstruction and not only their presence around the implant. This might be important for macrophages whose presence is routinely examined in the tissue surrounding an implant, which exist in various activation phenotypes and can be responsible for inflammation (M1 macrophages) or tissue remodeling (M2 macrophages). The number of M1 [33,34,35,36] macrophages present in the tissue surrounding implants is sporadically determined by immunohistochemistry, but antibodies used for detection (CD11c, CD80 and CD68 for M1 and CD163 for M2 cells) are not exclusive markers for those types of cells [40]. Having this in mind, to evaluate the biocompatibility of ACP/ChOL/Se-coated titanium we used a different approach by examining the expression of a marker of M1 cells (inducible nitric oxide synthase/iNOS) and M2 cells (arginase1/Arg1), inflammatory mediators (interleukin-1β/IL-1β, IL-6 and tumor necrosis factor/TNF), a factor contributing to angiogenesis (vascular endothelial growth factor/VEGF) and fibrosis (transforming growth factor β/TGF-β) in the tissue surrounding the implant and compared them with the response in the tissue around pure titanium.

The goal of this research was in situ anodization/anaphoretic deposition of an ACP/ChOL/Se multifunctional hybrid coating on a titanium substrate and in vivo examination of the immunomodulatory and anti-inflammatory effect of the novel ACP/ChOL/Se hybrid coating on the titanium substrate that could potentially be used as medical implant. The effect of the novel coating on phenomena in the interface tissue–material was not examined by the traditional histological approach, but by the examination of functional aspects in the tissue around the implant: proinflammatory cytokines’ gene expression, M1 and M2 macrophages, fibrous capsule formation and vascularization.

## 2. Materials and Methods

### 2.1. Synthesis

Amorphous calcium phosphate (ACP) was obtained by rapid addition of 150 mL of a 26.6 mass% solution of Ca(NO_3_)_2_ in double-distilled water to 400 mL of (NH_4_)_3_PO_4_ solution. (NH_4_)_3_PO_4_ solution was obtained by mixing 7 mL of H_3_PO_4_, 165 mL of NH_4_OH and 228 mL double distilled H_2_O. The resulting solution was constantly stirred at 100 rpm and 50 °C for 60 min. Obtained fine precipitate (gel) was left to age for 15 s before being collected and then rinsed with water and centrifuged at 4000 rpm in a Hettich Universal 320 centrifuge at 5 °C for 1 h. The precipitate thus obtained was freeze-dried at −30 °C at a pressure of 0.37 bar for 1 h. The final drying was performed at −40 °C and a pressure of 0.12 bar for 2 h.

Titanium plates, 99.7% pure (ThermoFisher, Waltham, MA USA), dimensions (20 × 10 × 0.89) mm, were used for in situ anaphoretic deposition processes of ACP/ChOL/Se hybrid multifunctional composite coatings on Ti substrate. Titanium plates were sanded with silicon carbide (SiC) sandpaper with 600, 1000, 2000 and 3000 grit, after which the plates were polished using alumina with grain sizes of 1, 0.3 and 0.05 µm (Buehler, IL, USA). All samples were further washed and purified in 96% ethanol (Sigma Aldrich, Taufkirchen, Germany) in a ASONIC PRO 50 (ASonic, Ljubljana, Slovenia) ultrasonic cleaner (120 W power, 40 kHz) for 30 min. The samples were stored in ethanol prior to deposition in order to prevent spontaneous oxidation.

ACP/ChOL/Se hybrid coatings on Ti substrate were obtained by in situ anaphoretic precipitation from appropriate ethanolic suspension. Na_2_SeO_3_ in the amount of 273.5 mg (for a total of 125 mg Se, Sigma Aldrich, Taufkirchen, Germany) was added to 50 mL double distilled water and stirred for 5 min until it was completely dissolved. A total of 125 mg of chitosan oligosaccharide lactate (ChOL, Mw 5000, Sigma Aldrich, Taufkirchen, Germany) was added afterwards to the same 50 mL solution and constantly stirred at 300 rpm for 60 min on a rotary magnetic stirrer IKA C-MAG HS 4 (Staufen, Germany). The total amount of selenium (125 mg) and the mass ratio of ChOL:Se = 1:1 were chosen since the authors only wanted to test the idea and confirm the concept of immunomodulatory effect of the ACP/ChOL/Se hybrid coating. After the ChOL has swelled and dissolved for an hour, 50 mL of 96% ethanol (Sigma Aldrich, Taufkirchen, Germany) and 1.000 g of ACP were added to the solution and further stirred at 300 rpm for 60 min. pH was adjusted with 5 mL of 1M NaOH. NaOH solution was added in order to increase the stability of the suspension for the subsequent anodization/anaphoretic deposition process. The suspension was mixed on a rotary magnetic stirrer at 300 rpm in order to homogenize the particles in the suspension and maintain a stable suspension all the time during deposition. In situ anaphoretic deposition was performed in a custom made two-electrode electrochemical cell. The anode was a titanium plate measuring (20 × 10 × 0.89) mm, and a pair of 316 grade stainless steel plates, measuring (20 × 10 × 0.89) mm, were used as the cathode, placed in parallel in relation to anode at a distance of 10 mm. ACP/ChOL/Se hybrid coatings on titanium were deposited at a constant voltage of 60 V for 1 min. The resulting coatings were air-dried for 24 h at 25 °C.

The morphology of hybrid coatings’ surface was analyzed by field-emission scanning electron microscopy (Tescan Mira 3 XMU FEG-SEM). EDS analysis was performed on a Jeol JSM 5800 SEM with SiLi X-ray detector (Oxford Link Isis series 300, Abingdon, UK). Wide-angle X-ray scattering (WAXS) method was used. Fourier transform infrared spectroscopy (FTIR) was performed on Michelson MB Series Bomen FTIR spectroscope (Hartmann Braun, Munich, Germany) in the range from 400 to 4000 cm^−1^ with a resolution of 0.5 cm^−1^. X-ray measurements for structural and phase evaluation of the multifunctional hybrid composites were performed on Philips PW 1050 diffractometer at 25 °C. Scintillation detector within 2θ range of 3–90° in steps of 0.02° and Ni-filtered CuKα radiation (λ = 1.54178 Å) were used. The scanning rate was 5 s per step. Linear roughness of ACP/ChOL/Se hybrid coatings on Ti substrate was measured using roughness tester TR-200 (Innovatest, Maastricht, The Netherland).

### 2.2. Animal Implantation

All animal treatments and procedures were performed in compliance with the Directive EU (86/609/EEC) on the care of animals used for experimental and other scientific purposes. Moreover, they were approved by Veterinary Directorate, Ministry of Agriculture, Forestry and Water Management (No. 323-07-10702/2022-05). Dark Agouti (DA) 10–12 weeks old male rats, conventionally housed and bred at the Institute for Biological Research ‘‘Siniša Stanković’’, University of Belgrade, under controlled conditions (12 h photoperiod, 21–24 °C temperature and relative humidity of 60%) were used in experiments. The animals were anesthetized by intraperitoneal injection of 10 mg/kg b.w of Zoletil 100 (Virbac, Carros, France). The hair of the dorsum was clipped off, and an incision was made in the scapular region, followed by a blunt incision to create a subcutaneous pocket. Material (1 × 1 cm), previously sterilized under UV-light for 20 min, was implemented under aseptic conditions. Animals were euthanized by Zoletil overdose at days 7, 14 and 28 following implantations, and the tissue surrounding the implant was collected. For each time point, 8 animals were assigned for ACP/ChOL/Se-coated and pure titanium implants.

### 2.3. Isolation of RNA, Reverse Transcription and Real-Time Polymerase Chain Reaction (RT-PCR)

Collected tissue samples were immediately homogenized in mi-Total RNA Isolation Kit (Metabion, Martinsried, Germany) according to manufacturer’s recommendation. Isolated RNA (1 µg) was reversely transcribed using random hexamer primers and MMLV (Moloney Murine Leukemia Virus) reverse transcriptase (Fermentas, Vilnius, Lithuania), following manufacturer’s instructions. Obtained cDNAs were amplified using Power SYBR^®^ Green PCR Master Mix (Applied Biosystems, Foster City, CA, USA) based on the recommendations of the manufacturer in a total volume of 20 μL in an ABI PRISM 7000 Sequence Detection System (Applied Biosystems). Thermocycler conditions consisted of an initial step at 50 °C for 5 min, followed by a step at 95 °C for 10 min and a subsequent 2-step PCR program at 95 °C for 15 s and 60 °C for 60 s for 40 cycles. PCR primers (forward/reverse) used in this study are listed in Table 1. PCR results were analyzed with 7500 System software (Applied Biosystems) and calculated as 2^−dCt^, where dCt was the difference between the threshold cycle (Ct) values of the specific gene and the endogenous control (β-actin).

### 2.4. Data Display and Statistical Analysis

Data from in vivo study are presented as mean (±standard deviation) from 8 animals per group per time point. For comparison between ACP/ChOL/Se-coated and pure titanium implants, Mann–Whitney U-test was used (STATISTICA 7.0, StatSoft Inc., Tulsa, OK, USA), and *p*-values less than 0.05 were considered significant.

## 3. Results

**Figure 1:**
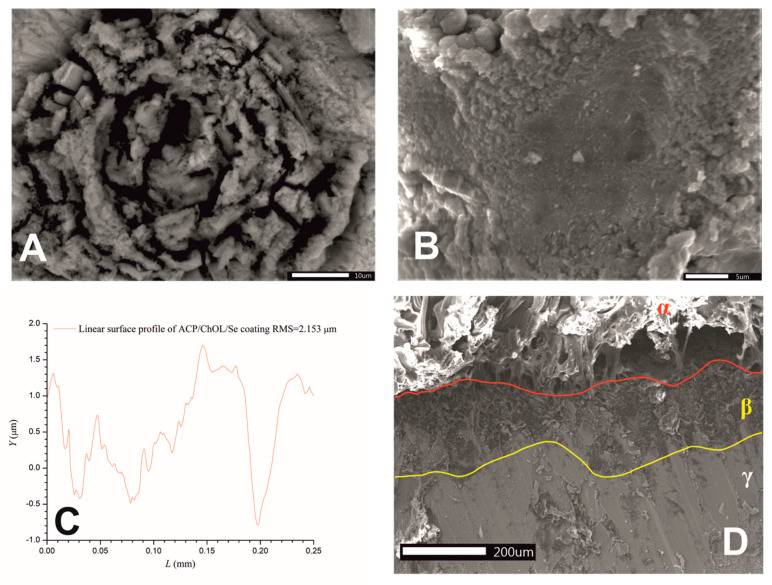
SEM images of ACP/ChOL/Se hybrid coating on titanium substrate deposited by in situ anodization/anaphoretic deposition process at 60 V (**A**) after 10 s of deposition and (**B**) after 1 min of deposition. (**C**) Linear roughness measurements and (**D**) SEM of cross-section of ACP/ChOL/Se hybrid coating on titanium (α—epoxy resin during sample preparation, β—hybrid coating, γ—Ti substrate). Image sizes are annotated by the scale bar in the lower right corner (Figure 1A,B) and in the lower left corner (Figure 1D).

SEM imaging was used to characterize physical appearance and surface area of synthesized hybrid ACP/ChOL/Se coatings on titanium substrates. Figure 1A shows the ACP/ChOL/Se coating at the beginning of the in situ anodization/anaphoretic deposition process (in the first 10 s), while Figure 1B shows the ACP/ChOL/Se hybrid coating after the in situ process is finished (after 1 min). Figure 1C shows measurements of linear roughness, namely the root mean square roughness (RMS) results. RMS represents the square root of the arithmetic mean of the squares of profile deviation from mean within the sampling length. A cross-sectional SEM image of the ACP/ChOL/Se hybrid coating is presented in Figure 1D.

**Figure 2:**
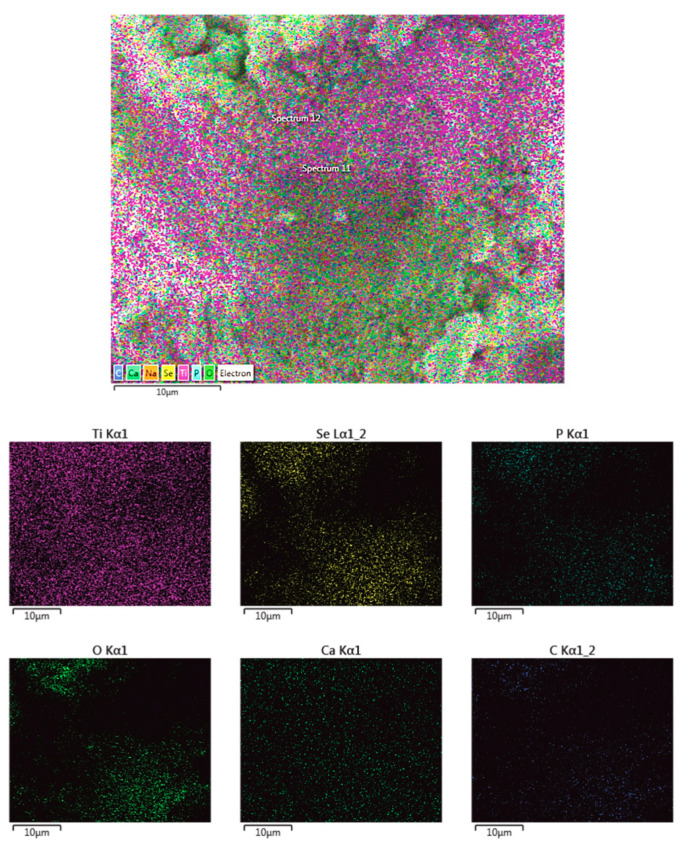
EDS mapping of ACP/ChOL/Se hybrid coating on titanium substrate deposited by in situ anodization/anaphoretic deposition process at 60 V after 1 min of deposition. Image sizes are annotated by the scale bar in the lower left corner of the respective image.

Figure 2 shows the EDS mapping of the ACP/ChOL/Se hybrid coatings on the titanium substrate, while Table 2 shows surface EDS measurement results in at.%.

**Figure 3:**
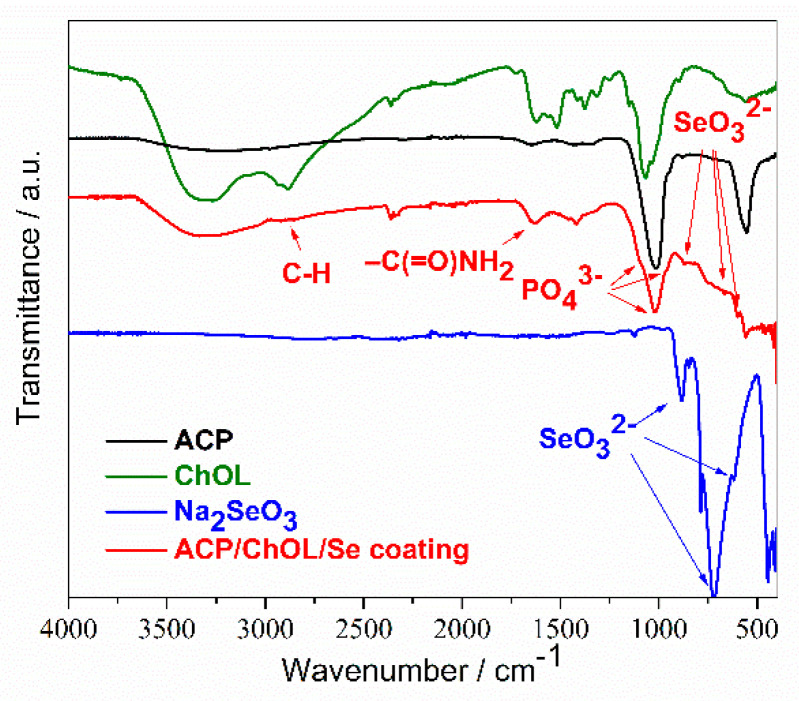
FTIR spectra of ACP (black line), ChOL (green line), Na_2_SeO_3_ powder (blue line) and ACP/ChOL/Se multifunctional hybrid composite coating (red line) on titanium substrate deposited by in situ anodization/anaphoretic deposition process at 60 V after 1 min of deposition.

The results of the FTIR characterization of Na_2_SeO_3_ powder and the ACP/ChOL/Se hybrid coating on the titanium substrate deposited by in situ anodization/anaphoretic deposition process at 60 V are presented in Figure 3.

**Figure 4:**
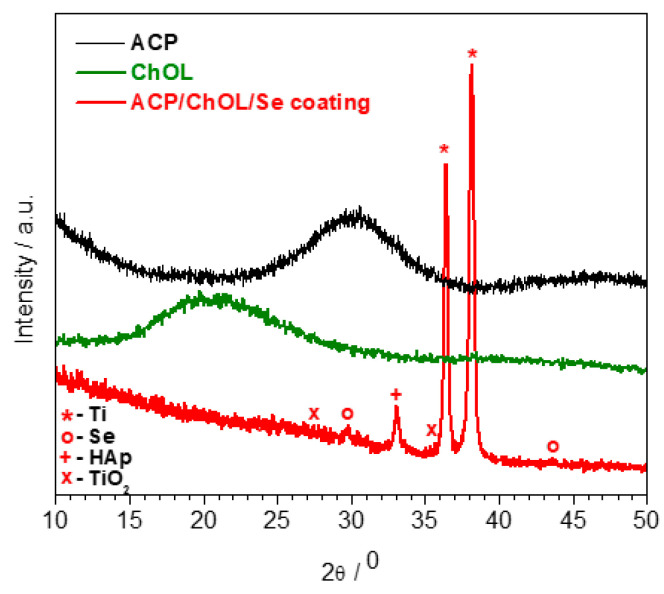
XRD diffractogram of ACP (black line), ChOL (green line) and ACP/ChOL/Se (red line) hybrid coating on titanium substrate deposited by in situ anodization/anaphoretic deposition process at 60 V after 1 min of deposition.

Figure 4 displays the XRD diffraction patterns of ACP, ChOL and ACP/ChOL/Se hybrid coating on the titanium substrate.

### In Vivo Study

To examine the effect of the novel coating on biocompatibility, we did not use the traditional approach (histological examination) but decided to examine functional aspects in the tissue around the implant that might give better insight into the presence of inflammation (proinflammatory cytokines’ gene expression), M1 macrophages (iNOS), M2 macrophages (Arg1), fibrous capsule formation (TGF-β) and vascularization (VEGF).

**Figure 5:**
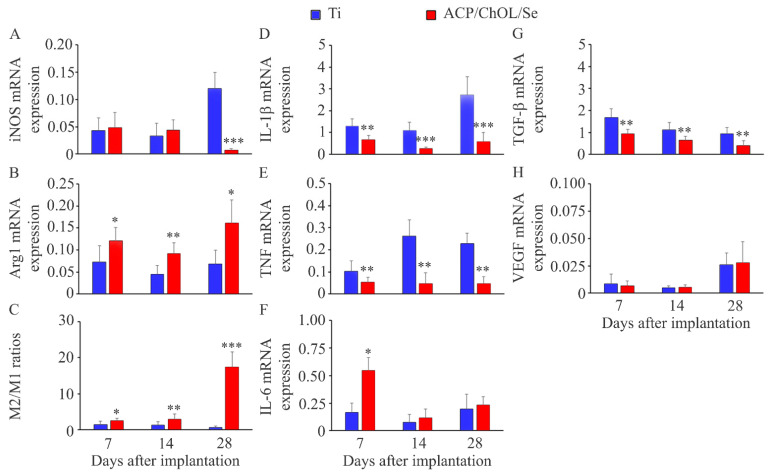
Gene expression in tissue surrounding pure titanium and ACP/ChOL/Se-coated titanium disks following subcutaneous implantation in rats evaluated by RT-PCR analysis. (**A**) mRNA expression of M1 macrophage marker iNOS. (**B**) mRNA expression of M2 macrophage marker Arg1. (**C**) M2/M1 ratio calculated as Arg1/iNOS. mRNA expression of IL-1β (**D**), TNF (**E**), IL-6 (**F**), TGF-β (**G**) and VEGF (**H**). Data are presented as mean ± standard deviation from 8 animals per group per time point. Statistically significant differences at * *p* < 0.05, ** *p* < 0.01 and *** *p* < 0.001 for ACP/ChOL/Se-coated titanium vs. pure titanium disks.

The ACP/ChOL/Se coating of the titanium implants did not affect the general physical condition of the animals, and there was no evidence of redness, swelling or infection around the implanted disks. The examination of macrophage functional polarization showed a similar presence of M1 macrophages (iNOS expression) (Figure 5A) at earlier time points, but lower at day 28 post-implantation in the tissue around the ACP/ChOL/Se implants. Additionally, a higher level of M2 polarization (Arg1 expression) (Figure 5B) was noted at all time points examined, resulting in a higher M2/M1 macrophage ratio (Figure 5C) in the ACP/ChOL/Se-coated implants compared to pure titanium implants. Lower inflammation measured by gene expression of proinflammatory cytokines IL-1β (Figure 5D) and TNF (Figure 5E) was noted in presence of the ACP/ChOL/Se implants at all time points examined. In contrast to IL-1β and TNF, a transiently higher IL-6 expression (solely at day 7 post-implantation) (Figure 5F) was observed in the ACP/ChOL/Se implants. The tissue surrounding the ACP/ChOL/Se implants was characterized by a lower expression of TGF-β (Figure 5G) at all time points post-implantation, while no differences were detected in VEGF expression (Figure 5H).

## 4. Discussion

Two distinct and morphologically different surfaces can be observed in Figure 1A,B. Figure 1A shows a rose flower-like structure of the deposited coating. This feature can be explained by the mechanism of the in situ process. Unlike the previous coatings [9,13], the suspension for anodization/anaphoretic deposition of ACP/ChOL/Se hybrid coatings has more conductive species (Se-containing ions). Since the deposition is performed under potentiostatic conditions, the current is much higher (at the beginning of the process it is one order of magnitude greater than for the previous experiments), and the process leads to hydrogen evolution. This gas evolution leads to formation of pits, holes and vacancies that form rose flower-like structure. As the process continues, more and more material covers the substrate, more material is deposited as a composite coating. The conductivity of the working electrode decreases as the resistivity of the overall system increases, i.e., the system resistivity approaches the one that was for the ACP/TiO_2_/ChOL system. The current decreases by one order of magnitude. From Figure 1B, it can be noticed that the coating completely covers the surface of the titanium substrate, and that the coating is made of agglomerated nanoparticles. One should bear in mind that the starting particles are smaller than 100 nm, as shown in our previous research [13]. The agglomerates form coarse surface, which is prerequisite for good osteoconductivity.

From Figure 1C, it can be seen that the surface of the hybrid ACP/ChOL/Se coating on the titanium substrate has more pronounced smaller profile shapes. The RMS value for the hybrid ACP/ChOL/Se coating is 2.153 µm, and the measurements are comparable and in consistence with the previously published results [13]. Figure 1D presents a compact hybrid ACP/ChOL/Se coating, which has a thickness of 240 ± 25 μm, and it is labeled as β on Figure 1D. The coating structure and thickness are completely in accordance with the previous research by the authors [13]. Value γ belongs to the Ti substrate, and the top layer, labeled as α, belongs to the epoxy resin used to protect the coating while it was cross-cut for the analysis.

It can be seen from Figure 2 that the coating is of homogenous structure. All of the components, namely, ACP (Ca and P), chitosan lactate polymer (C) and Se additive (Se), are uniformly distributed on the titanium (Ti) substrate. The EDS measurements of the ACP/ChOL/Se hybrid coating that are presented in Table 2 show elements that confirm the presence of amorphous calcium phosphate, namely Ca/P ratio (which is 1.67). The presence of C in the measurements show the presence of chitosan oligosaccharide lactate, while the presence of selenium is also confirmed. Oxygen is present in the polymer, oxidized titanium and used selenium salt, while the presence of sodium is explained by the use of sodium selenite.

The FTIR measurements presented in Figure 3 show characteristic absorption bands that correspond to PO_4_^3−^ group from ACP (distinguishable peak at 1019 cm^−1^ with two shoulders at 960 cm^−1^ and 1195 cm^−1^) [9]. Moreover, a strong adsorption band at 1628 cm^−1^ (assigned to the amide I band) along with a weak adsorption band at around 2923 cm^−1^ (attributed to –C-H backbone vibrations) belong to the ChOL polymer [9]. Finally, adsorption bands at 620 cm^−1^ and 719 cm^−1^ come from ν3 and ν1 stretching vibrational mod of SeO_3_^2−^, while the peak at 883 cm^−1^ belongs to δ in-plane bending of SeO_3_^2^ [41,42]. The former statement is also proven by comparing the blue FTIR spectrum from Figure 3 of pure Na_2_SeO_3_ powder to the red spectrum of ACP/ChOL/Se.

In Figure 4, diffraction peaks (o) at 2θ of 29.7° and 43.6° can be assigned to the (101) and (102) crystal latices of Se [43,44]. Other peaks have already been explained [9,12,13], and they belong to the Ti substrate and TiO_2_ phases. The most intensive peaks of the pattern were the Ti peaks (*) of the substrate (JCPDS standard XRD card No. 89-5009) at 2θ = 36.4 and 38.1°. The specific lowest intense XRD reflections of TiO_2_ (x) at 2θ = 27.8° and 36.5° (JCPDS standard XRD card No. 88-1173) are also seen. The peaks at around 32° (+) originate from ACP and in accordance with our previous investigations [9,12,13] are attributed to the transformation of ACP into its more ordered structure which is designated as low-crystalline hydroxyapatite (HAp).

### Results of In Vivo Study

Macrophages have an important role in the immune response to implants [45], and the presence of these cells in the tissue surrounding implants has been examined by histology or immunohistochemistry [33,34,35,36,46]. The ACP/ChOL/Se implant has no effect on M1 cells (measured by expression of the signature molecule for these cells) at earlier time points post-implantation, which might be beneficial as inflammation in early stages is important for the prevention of infection.

However, at later time points the ACP/ChOL/Se implant decreased the number of inflammatory cells suggesting better control of inflammation. An increased expression of Arg1 indicates that the ACP/ChOL/Se coating results in a higher differentiation of macrophages toward the M2 phenotype which is involved in tissue repair. A higher number of M2 macrophages and increased M2/M1 ratio has been documented for some implanted materials [33,34], and attempts exist to modulate macrophage response to implant materials toward M2 phenotype [46]. In this context, a higher number of M2 cells and M2/M1 ratio might indicate that the ACP/ChOL/Se coating has a beneficial effect on the immune response at the host–implant interface. A lower expression of IL-1β and TNF around the ACP/ChOL/Se implants indicates that selenium, although has no effect on M1 cell numbers, decreases the activity of these cells. Both IL-1β and TNF are produced by M1 macrophages [45] and are increased in response to various implanted materials [47] or titanium particles [48]. As these cytokines can activate osteoclastogenesis leading to osteolysis [48], lower levels of IL-1β and TNF induced by the ACP/ChOL/Se coating might be beneficial for implant integration. In contrast to a decreased IL-1β and TNF response to ACP/ChOL/Se-coated disks, a transient higher IL-6 response was noted in the presence of selenium. Although decreased osteolysis was noted following prolonged (4 weeks) neutralization of IL-6 in animals [48], early and transient production might be beneficial for tissue regeneration as this cytokine is a key modulator of the inflammatory and reparative processes [49]. A lower expression of TGF-β in the tissue around the ACP/ChOL/Se implants suggests reduced fibrous capsule formation in comparison to pure titanium, as a positive correlation between TGF-β and fibrosis progression has been documented [50]. Additionally, the lower expression of TGF-β might contribute to a better implantation/higher stability of ACP/ChOL/Se-coated titanium into the tissue. Supporting this assumption are the data showing a lower expression of this factor in the stromal cells, epithelial layers and in vascular component in mucosa around healthy dental implants compared to failing implants [51]. Formation of novel blood vessels is also important for tissue integration of medical devices, but neovascularization has been sporadically documented in papers examining the biocompatibility of tissue implants [33,34,35]. VEGF is a factor involved in the regulation of angiogenesis during tissue healing, and the data showing failing of dental implants in the patient under the treatment with the VEGF inhibitor [52], as well as a lower expression of this molecule in mucosa around failing implants compared to healthy implants [53], indicate an important role of VEGF in the process of tissue integration. Beneficial effects of VEGF have been shown in an animal model where a higher number of endothelial cells and osteoblasts around VEGF-coated implant (compared to control implants) were noted [54]. The results we obtained indicate that the ACP/ChOL/Se coating does not affect vascularization, as a similar expression of VEGF was noted in the tissue around both the ACP/ChOL/Se-coated and pure titanium disks.

Altogether, lower inflammation and fibrosis with a higher M2/M1 macrophage ratio and similar vascularization indicate that the ACP/ChOL/Se coating improves implant performance and might contribute to a higher stability of titanium implants.

## 5. Conclusions

In this work, in situ anodization/anaphoretic deposition process was used for the synthesis of an ACP/ChOL/Se multifunctional hybrid coating on a titanium substrate, and in vivo investigation of the immunomodulatory and anti-inflammatory effect of such implants was conducted. The SEM analysis of the coating morphology indicated that during the deposition process at the beginning, there is a rose flower-like structure formation due to hydrogen evolution, and the pits, holes and vacancies fill up while the process is progressing. This morphology change occurs due to the increase in the system resistivity, since less electroconductive coating is forming on the substrate surface. The EDS, FTIR and XRD analyses have proven the formation of an ACP/ChOL/Se multifunctional hybrid composite coating on Ti and the presence of Se.

The ACP/ChOL/Se implant has no effect on M1 cells (macrophages) at earlier time points post-implantation, which might be beneficial, as inflammation in early stages is important for the prevention of infection. At the same time, it was found that Arg1 and M2/M1 ratio expression increased, which might indicate that the coating has a beneficial effect on the immune response at the host–implant interface. A lower expression of inflammatory mediators (IL-1β and TNF) around the ACP/ChOL/Se implants indicates that selenium might be beneficial for implant integration. A transient higher IL-6 response (a key modulator of the inflammatory and reparative processes) was noted in the presence of selenium, and early and transient production might be beneficial for tissue regeneration. A lower expression of TGF-β in the tissue around the ACP/ChOL/Se implants suggests reduced fibrous capsule formation in comparison to pure titanium. Hence, the lower expression of transforming growth factor (TGF-β) might contribute to a better implantation/higher stability of ACP/ChOL/Se-coated titanium into the tissue. The obtained results indicate that the ACP/ChOL/Se coating does not affect vascularization. Lower inflammation and fibrosis with higher M2/M1 macrophage ratio and similar vascularization indicate that the ACP/ChOL/Se coating improves implant performance and might contribute to a higher stability of titanium implants.

The results unequivocally confirm that the ACP/ChOL/Se multifunctional hybrid composite coating on a titanium substrate has the immunomodulatory and anti-inflammatory effect compared to the pure grade 2 titanium implants that are widely used in medicine and dentistry. The proven concept leads the authors to the continuation of the research, where different concentrations of selenium, different active agents and pure calcium phosphate/polymer coatings on a titanium substrate will be tested and optimized. However, these studies are ongoing, and they are the subject of further research and future publications.

## Figures and Tables

**Table 1:** PCR primers used in this study.

Gene	Forward	Reverse
β-actin *	5′−CCCTGGCTCCTAGCACCAT-3′	5′-GAGCCACCAATCCACACAGA-3′
IL-1β	5′-CACCTCTCAAGCAGAGCA-3′	5′-GGGTTCCATGGTGAAGTCAAC-3′
IL-6	5′-CCCTTCAGGAACAGCTATGA-3′	5′-TGTCAACAACATCAGTCCCAAG-3′
TNF	5′-TCGAGTGACAAGCCCGTAGC-3′	5′-CTCAGCCACTCCAGCTGCTC-3′
iNOS	5′-TTCCCATCGCTCCGCTG-3′	5′-CCGGAGCTGTAGCACTGCA-3′
Arg1	5′-TGGACCCTGGGGAACACTAT-3′	5′- GTAGCCGGGGTGAATACTGG-3′
VEGF	5′-GGGCCTCTGAAACCATGAACT-3′	5′-ACGTCCATGAACTTCACCACTTC-3′
TGF-β	5′-CCCTGCCCCTACATTTGGA-3′	5′-ACGGTGATGCGGAAGCAC-3′

* Housekeeping reporter gene.

**Table 2:** Elemental analysis of the synthetized coating on Ti supstrate.

Elemental Analysis, at.%	C	O	Na	P	Ca	Ti	Se	Total
Hybrid ACP/ChOL/Se	9.32	28.10	2.11	3.64	6.10	34.98	15.75	100.00

## Data Availability

Data are available on request from the corresponding authors due to privacy restrictions.
